# Association of viral load and physical status of HPV-16 with survival of patients with head and neck cancer

**DOI:** 10.3332/ecancer.2020.1082

**Published:** 2020-07-30

**Authors:** Dayahindara Veitía, Juan Liuzzi, Maira Ávila, Idamelys Rodriguez, Felix Toro, María Correnti

**Affiliations:** 1 Molecular Genetics Laboratory, Oncology and Haematology Institute, Ministry of Popular Power for Health (MPPS), 1053, Venezuela; 2 Head and Neck Service, “Padre Machado” Oncology Hospital, Ministry of Popular Power for Health (MPPS), 1053, Venezuela; 3 Molecular Biology Laboratory, Immunology Institute, University of Central Venezuela (UCV), 1053, Venezuela

**Keywords:** head and neck, HPV, viral load, integration, survival

## Abstract

**Introduction:**

Head and neck cancers (NHCs) are of multifaceted origins, and tobacco and alcohol are the primary risk factors. Currently, other factors associated with the genesis of these tumours are being considered, among these viral infections, especially human papillomavirus (HPV) infection.

**Objective:**

The objective was to evaluate HPV infection, HPV-16 E6 load and its physical status in patients with squamous cell carcinoma in the head and neck and evaluate its effects in the survival of these patients.

**Methodology:**

A total of 80 fresh biopsies of HNC were evaluated. The genetic material was extracted using the commercial kit QIAGEN. The detection and classification of HPV were carried out using INNO-LiPA, whereas the quantification and analysis of integration of the viral genome into the host cell were carried out using real-time PCR.

**Results:**

The average age of the patients included was 60.34 ± 14.48 years, with a predominance of the male gender. The most frequent HPV infection was genotype 16 (52.8%), with an average of 10 copies of the HPV-16 E6/β-globin gene. Furthermore, an integration of the viral genome in the host cell was observed in 86% of cases with a statistically significant relationship between the location of the tumour and the viral load (*p* < 0.05).

**Conclusions:**

HPV-16 is the most common infection, and its physical status in the host cell is the determining factor in establishing response to treatment. However, more studies are needed to demonstrate the role of HPV infection in carcinogenesis.

## Introduction

Head and neck tumours of any oncological pathology are of multifactorial origin, and tobacco and alcohol are the primary risk factors. It is estimated that between 85% and 90% of cases of head and neck squamous cell carcinomas (HNSCC) are explained by exposure to these factors; the risk is being proportional to the intensity of the exposure [1]. In recent decades, viral infections have been suggested as the risk factors associated with the development of this malignity. It has also been seen that the presence of oncogenic viruses and their interaction with the genetic material of the host provoke can trigger the alteration in the genes, which regulate the cellular cycle of the infected cells [2].

Currently, there is epidemiological and molecular evidence that human papillomavirus (HPV) is related to squamous cell carcinomas, such as in the case of cervical cancer [3] and oropharyngeal cancer [4]. This infection, probably acquired from sexual contact, could alter the epidemiology and demographics of cancer of the aerodigestive tract. In consequence, a diagnosis of squamous cell cancer positive for HPV could be clinically relevant for prognostic purposes, as well as having future diagnostic and therapeutic implications, including its prevention [5]. The numerous molecular studies suggest that the high-risk oncogenic HPV types, especially genotypes 16 and 18, are aetiologically related to a subgroup of head and neck cancers, especially oropharyngeal squamous cell carcinomas. This indicates that HPV infection in the oropharynx could lead to a predisposition to tumourogenesis [4, 6, 7, 8]. It has been possible to determine the physical status of HPV in the host cells using enlargement techniques such as real-time PCR, considering that it is integrated when the E2 gene breaks off. It is found in its episomal form when there are a number of copies equivalent to the E2 and E6 genes. It is also described as a mixed form, where there are fewer copies of the E2 gene than of E6 [9]. This is of great importance since it has been considered that the integration of the viral genome into the cellular genome is an important step to consider in the malignant transformation; the breaking off of gene E2, which controls the expression of genes E6 and E7, results in an increase in the expression of said oncogenes. E6 is related to the degradation of P53, whereas E7 with the degradation of the retinoblastoma protein (pRB); both are important in regulating the cell cycle [6].

Driven by everything set out previously, the objective of this investigation is to evaluate HPV infection, HPV-16 E6 load and its physical status in patients with squamous cell carcinoma in the head and neck and evaluate its effects in the survival of these patients.

## Materials and methods

### Biological material

A total of 80 fresh biopsies from HNSCC tumours were evaluated. These were gathered by Dr Juan F. Liuzzi of the Head and Neck Service in the Oncology Hospital ‘Padre Machado’ in Caracas.

Each patient included in this study had to give their approval by signing an informed consent as well as responding to a survey, which can be found as part of the FONACIT projects G-2005000408, 2013000413 and Misión Ciencia (Science Mission) LPL-20074001088.

### Extraction of the genetic material

The extraction of the nucleic acids was carried out using the commercial DNA Mini Kit QIAGen (250) following the firm’s specifications.

From each biopsy, a piece of tissue approximately 25 mg was cut, and this, in turn, was cut into small pieces and incubated in ATL buffer (180 μL) and 20 μL of proteinase K overnight with agitation at 56ºC. Afterwards, 200 μL of buffer AL was added, vortex mixed for 15 sec and incubated at 70ºC for 10 min.

200 μl of absolute ethanol was added, and then, it was transferred to a tube and centrifuged at 6,000 xg for 1 min; Nearly 500 μL of the buffer of washed AW1 was added and centrifuged at 6,000 xg for 1 min; 500 μL of the buffer of AW2 was added and centrifuged at 20,000 xg for 3 min. Then, 200 μL of AE buffer was added, and it was incubated at room temperature for 1 min and centrifuged at 6,000 xg for 1 min. The genetic material gathered was stored at −80ºC until its subsequent use.

### Detection and genotyping of the human papillomavirus

HPV detection was performed using the INNO-LiPA HPV Genotyping Extra Kit (Innogenetics), following the commercial specifications. The kit allows specific detection of 25 HPV genotypes (HPV types 6, 11, 16, 18, 31, 33, 35, 39, 40, 42, 43, 44, 45, 51, 52, 53, 54, 56, 58, 59, 66, 68, 70, 73, 74), and it is based on reverse hybridisation. The test included a PCR amplification of a 65 bp fragment within the L1 region of the HPV genome using the broad-spectrum SPF10 biotinylated primers. Biotinylated amplicons are subsequently hybridised with HPV type-specific oligonucleotide probes which are immobilised as parallel lines on membrane strips. After hybridisation and stringent washing, streptavidin-conjugated alkaline phosphatase is added and bound to any biotinylated hybrid formed. Incubation with BCIP/NBT chromogen yields a purple precipitate, and the results can be visually interpreted.

### Quantification and evaluation of the physical status of the HPV-16 genome

The quantification of HPV-16 E6 as well as evaluation of the genome’s integration was carried out using real-time PCR according to the specifications described by Koskinen et al. [9].

Real-time PCR was performed with the 7500 Real-Time PCR System (Applied Biosystem) in the Molecular Biology Laboratory at the Immunology Institute of the Central University of Venezuela. The sequences for the initiators are shown in Table 1. The amplification and quantification of the E6 and E2 genes were carried out simultaneously in separate reaction tubes. The reaction mixture consisted of 50 *μ*L made up of 1X SSoFast Evagreen (BIORAD), 900 nM of each of the initiators and approximately 50 ng of the DNA. The amplification process consisted of 1 cycle at 95°C for 10 min, 44 denaturing cycles at 95°C for 15 sec and the binding and extension phase at 60°C for 1 min, followed by the dissociation curve.

All the samples that tested positive for HPV genotype 16 were evaluated to check whether gene E2 had broken off, as well as quantifying genes E2 and E6 of HPV genotype 16.

The calibration curve was performed using DNA from HeLa cells, the initiators GP5+/GP6+ were used, which allow for the detection of the HPV genome, and the initiators PC04 and GH20 were used to amplify the β-globin human gene (shown in Table 1). The extracted DNA was quantified (Beckman Instruments DV series Spectrophotometer. 260–280 nm). Afterwards, the serial dilutions of this material were made which were used for the respective qRT-PCR. For HPV, 1.5 *μm of each initiator was used, 1X SsoFast Eva Green in a final volume of 20 μ*L. For the amplification and quantification of the β-globin gene, 1 *μM of the initiators PC04 y GH20, 2X SsoFast Evagreen were used in a final volume of 50 μl.* The amplification process used was 95°C followed by 40 cycles at 95°C, hybridisation at 46°C for 1 min and extension at 72°C over 30 sec. Once the amplification processes had been performed both for the standard curve and the viral load of the E6 and E2 genes, electrophoresis was carried out in a 2% agarose gel dyed with ethidium bromide (0.2 μL of a solution of 1%) and exposed to UV light on an imaging camera ChemiDoc™ XRS+ (Bio-Rad) as a photographic record, corroborating that the process had been carried out successfully.

### Statistical analysis

The relationship between the viral infection and the development of tumours was evaluated using the Chi-square test (*X*^2^), and the statistical significance was considered for values of *p* < 0.05.

For the analysis and estimation of the patient survival curves, the Kaplan–Meier method was used. The statistical significance was considered for values of *p* < 0.05.

In both the cases, the statistical program IBM SPSS Statistics 25 was used.

## Results

The average age of the patients was 60.34 ± 14.48 years (range: 19–89). The most frequent gender was males with 68.75% (*p* = 0.001). Regarding the associated risk factors, 73.5% (*p* = 0.000) of the patients were smokers and 70% consumed alcohol (*p* = 0.000). The most frequent location of the tumour was the oral cavity (33.8%), followed by the larynx (30%) and the oropharynx (22.5%). In addition, the paranasal sinuses (5%), nasopharynx (3.8%), hypopharynx (2.5%) and nasal cavities (2.5%) are also presented as the locations of the primary tumour (Table 2).

Regarding the histopathological analysis of the tumours, 40% corresponded to extensive tumours (T4) and moderately differentiated (43.75%), in the advanced states of the illness (EIII and EIV).

Sixty-six percent of the samples evaluated were positive for HPV infection (*p* = 0.004), and HPV-16 was the most frequent form of single infection (17%). It could be noted that the infection for this viral genotype was present in the form of a coinfection or mixed infection in a total of 52.8% of those testing positive for HPV-16 (Table 3).

Regarding the presence of the infection according to the anatomical location of the primary tumour, it was observed that the oral cavity, larynx and oropharynx were the most frequent with 34%, 28% and 23%, respectively; the majority of the HPV-positive tumours were found in advanced states EIII (26%) and EIV (55%) (Table 3). Of these patients, 60% were smokers and 58% consumed alcohol (data not shown).

The average viral load was 10 copies of DNA of HPV for each copy of β-globin (range between 2 and 20 copies of DNA of HPV), and the oropharynx was the anatomical location with the largest number of copies. At this site, with an elevated copies number of E6-HPV-16, the viral genome was found in an episomal or mixed form. The oral cavity and the larynx, on the contrary, presented low copies numbers of E6-HPV-16, and in these, the viral DNA was found in an integrated form (*p* = 0.00) (Table 4). The average frequency of integration was 86%; a statistically significant relationship was observed between the low viral load and the anatomical location with integration (*p* = 0.016).

**Figure 1.**
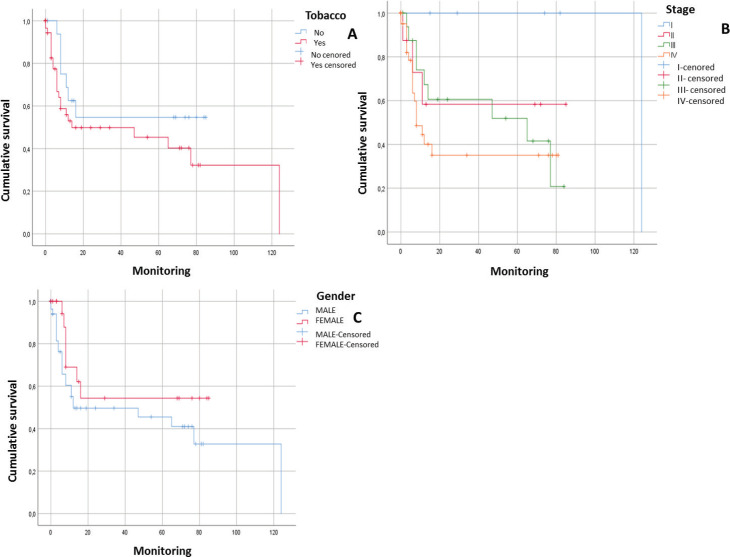
Survival curves of patients with squamous cell carcinoma in the head and neck. A) Patients with HNSCC in relation to tobacco consumption (*n* = 80; *p* = 0.187). B) Patients with HNSCC in relation to tumour stage (*n* = 80; *p* = 0.087). C) Patients with HNSCC in relation to gender (*n* = 80. *p* = 0.171).

**Figure 2.**
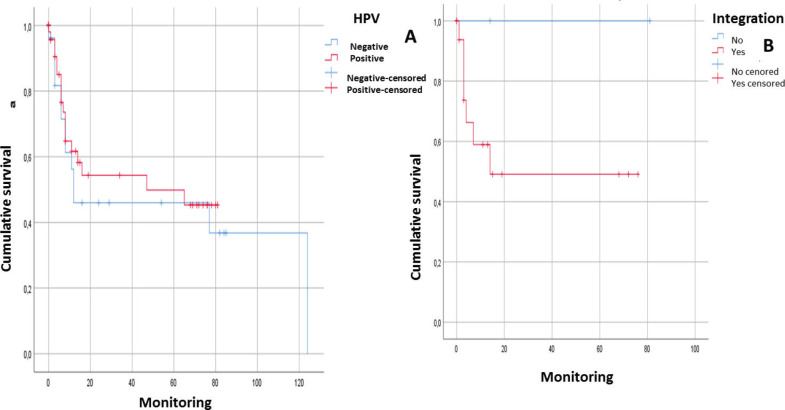
Analysis of survival of patients with squamous cell carcinoma in the head and neck in relation to: A) HPV genome presence (without distinguishing genotype) (*n* = 53; *p* > 0.05). B) Integration of the viral genome into the host cell genome (*n* = 28. *p* > 0.05).

After following the patients for 120 months, it was observed that there was a tendency towards the reduction in the survival of the male patients, there was some engagement in other risk behaviours (alcohol and/or tobacco) and they were diagnosed in advanced stages. In addition, it could be noted that the physical status of the viral genome in the host cell played an important role; in those cases, when the viral DNA presented in an episomal form, they had a more constant survival than those patients whose viral DNA presented in an integrated form although without a statistically significant relationship (*p* > 0.05) (Figures 1 and 2).

## Discussion

HNSCC presents primarily in males, and the age of appearance is being an important factor [10, 11]. In this investigation, the average age was maintained within the range described of 60.34 ± 14.48 years (range 19–89), and in agreement with the previous reports, this type of malignity presents most frequently between the fifth and seventh decades of life, with a mean average of 62 ± 12 years [12].

Sixty-six percent of the samples evaluated were positive for the presence of the HPV genome, this value is higher than that reported by the other works which report the viral detection averages of 39%–40% [13, 14]**,** 60% according to Yang et al. (2019) [15] and even 61% according to Koskinen et al. [9]; however, it remains within the averages reported by the other authors [16, 17]. Regarding genotype, HPV-16 was the most frequent in single infection form (17%), noting that the infection in this genotype be it in the form of co-infection or mixed infection was present in a considerable number of the analysed cases. This is within the ranges described by Koskinen *et al* [9] and Tao *et al* [18], who reported 27% and 85% positivity for HPV, respectively. Another important aspect which could be noted was that, in initial stages of the illness, the frequency of HPV infection is low (4%), and this increases as the illness stage advances (55% in advanced stages). This supports the claims carried out in other anatomical areas such as the cervix, indicating that a persistent infection with high-risk oncogenic genotypes, amongst them HPV-16, is an important risk factor for the genesis and progression of a tumour [19]. In addition, 60% of patients with HPV engage in other risk factors such as alcohol and tobacco, suggesting that these could also act as the risk factors for HPV infection and that combined (alcohol, tobacco, HPV and other infections) they could all act in a synergistic way in the process of development and progression of the malignity.

The oral cavity, larynx and oropharynx were, in this order, the anatomical locations most frequently infected with HPV. This is contrary to publications which describe the oropharynx as the area of the head and neck with the greatest infection rate [13]. However, the results of various investigations agree that these three areas are those most frequently infected by HPV. On the other hand, it suggests that the oral cavity is ‘the door of entry of the virus’, and this could coincide with an increased frequency of viral infection. It also suggests that HPV infections are a biological factor, which plays an important role in the carcinogenesis of these anatomical locations, based on epidemiological and clinicopathological evidence [20]. HPV-16 is being identified between 29% and 90% of these evidence [21–24]. It also proposed that the presence of this viral genotype in exfoliated cells of the oral cavity increases the risk of oropharyngeal cancer by 14 times [25].

On the contrary, the result of the average viral load investigation agrees with the previous works which explain that it can vary in these types of tumours and can be anywhere from 3.4 × 10^-4^ up to values as high as 739 copies/reference gene [9, 26]. If the results are compared with those of other anatomical locations, such as the oesophagus, for example, it could be said that the number of copies of HPV-16 E6 is similar, approximately 2.55 ± 3.19 copies per cell [27]. In this vein, Si *et al* [28] reported that, in China, the average viral load varies between ˂1 and 157 copies per cell in this anatomical location; as it can be seen, these are significantly low values if they are compared with those described for the cervix, vagina and vulva. whose reports reach values of more than 1 million copies of the HPV-16 E6 gene [27, 29].

As can be seen, viral load is not constant and presents notable differences between anatomical locations. It has been of great use evaluating the integration of the viral genome to the host cell genome since the previous studies reveal that the state of the viral genome is an important prognostic factor that, together with epigenetic factors, they are related with the cancer progression [30]. In this sense, it has been reported that those cases with episomal HPV show a favourable prognosis, suggesting that integration could, in some way, modify the biology of the tumours [31]. Eighty-six percent of the samples included in this study showed the integration of the viral genome with the host cell genome. This is consistent with other investigations, such as carried out by Olthof *et al* [32]; Otlthoft *et al* [33] and Mellin *et al* [31], who all indicate a high percentage of integration. Equally, they show that viral load is variable in HNSCC and that a high viral load is associated with the preferably episomal status, which is related with a significantly better prognosis, whereas low loads are related with integration and a poor prognosis. This was observed, in this study, in the cases of oral cavity and larynx tumours when the viral load was low, where the largest percentage of integration was detected. On the contrary, in the oropharyngeal tumours, the viral load was high in relation to the other anatomical locations evaluated, and the viral DNA was found in the preferable episomal form, which was related with a better prognosis (*p* = 0.016).

The investigations attribute these differences to the fact that the patients with high viral load possibly induce a better immune response against cancer although this hypothesis has not been confirmed and requires more study to support it. It is important to mention that although there are notable variations in the viral load between different anatomical locations, the average percentage of integration is similar to that described in the investigation carried out in the cervix by Groves and Coleman [34]. It is also important to mention that this process of integration requires the breaking of both the viral genome and the host cell’s genome, and it is believed that the rate of integration is proportional to the level of damage suffered by the DNA. These damages have been described as endogenous and exogenous, including the effects of alcohol and tobacco as well as HPV infection or other infectious agents, such as the Epstein–Barr virus (EBV), for example. All are the risk factors for the development of HNSCC, and this triggers an activation in the DNA repair mechanisms, as well as the process of chromosome alteration both of them could contribute to the process of integration [35].

After following the survival of the patients related to each one of the variables, for a period of 120 months, it could be noted that there was a downward trend in the survival of the male patients, something that was also noted in those patients who engage in risk activities such as consumption of alcohol or tobacco as well as those who presented with a diagnosis in an advanced stage (EIII and EIV). Equally, it was not possible to demonstrate that the presence of HPV infection would have a direct effect on survival. However, it would appear that an integration of the viral genome could be the determining factor since in those cases, where the HPV-16 genome was integrated, the survival was lower than in those cases, where it was in an episomal form. Although there was a statistically significant evidence to support this, this could be attributed to the low number of samples included (Figures 1 and 2).

## Conclusions

The oral cavity is the most common anatomical location for tumour growth, which could be due to it being in direct contact with aetiological agents. On the other hand, the presence of HPV-16 is established as the most common viral infection, considering the physical status of the viral genome as the determining factor for being able to establish the response to the employed therapies. It is suggested that HPV-positive patients, especially with oropharyngeal cancer, have a better prognosis than those who develop this type of cancer in other anatomical locations in the upper aerodigestive tract; these differences in prognosis could be due to the physical status of the viral genome in the host cell. However, the need to carry out more studies should be highlighted, to be able to evidence the role of HPV infection in carcinogenesis.

## Conflicts of interest

The authors have no conflicts of interest to declare.

## Figures and Tables

**Table 1.** Sequences of the initiators used in the real-time PCR to quantify the viral genome.

Initiator name	Sequence 5ʹ----› 3ʹ
HPV-16 E2F primer	ACGACTATCCAGCGACCAAGAT
HPV-16 E2R primer	CCACTGAGTCTCTGTGCAACAAC
HPV-16 E6F primer	GCACAGAGCTGCAAACAACTATACA
HPV-16 E6R primer	TCCCGAAAAGCAAAGTCATATACC
GP5+	TTTGTTACTGTGGTAGATACTAC
GP6+	CTTATACTAAATGTCAAATAAAAAG
PC04	CAA CTT CAT CCA CGT TCA CC
GH20	GAA GAG CCA AGG ACA GGT AC

**Table 2.** Characteristics of the samples in the study.

Sample characteristics	Percentage of patients (%)	*p*-value
**Gender**		
Female	31.25 (25/80)	0.001
Male	68.75 (55/80)	
**Smoking habits**		
Yes	73.75 (59/80)	0.000
No	26.25 (21/80)	
**Alcohol consumption**		
Yes	70 (56/80)	0.000
No	30 (24/80)	
**Primary tumour site**		
Oral cavity	33.8 (27/80)	0.000
Larynx	30 (24/80)	
Oropharynx	22.50 (18/80)	
Paranasal sinuses	5 (4/80)	
Nasopharynx	3.80 (3/80)	
Hypopharynx	2.50 (2/80)	
Nasal cavities	2.50 (2/80)	
**Tumour size**		
T1	8.80 (7/80)	
T2	21.2 (17/80)	
T3	30 (24/80)	
T4	40 (32/80)	
**Tumour grade**		
G1	20 (16/80)	0.03
G2	43.75 (35/80)	
G3	36.25 (29/80)	
**Tumour stage**		
EI	6.30 (5/80)	0.000
EII	12.50 (10/80)	
EIII	27.50 (22/80)	
EIV	53.8 (43/80)	

**Table 3.** HPV infection in patients with HNSCC.

Detection of HPV infection	Percentage of patients	*p*-value
Positive	66 (53/80)	0.004
Negative	34(27/80)	
**Genotype**		
16	52.8 (28/53)	0.000
Other LR genotypes**	20.7 (11/53)	
Other HR genotypes*	26.4 (14/53)	
**Anatomical location**		
Oral cavity	34 (18/53)	0.606
Larynx	28(15/53)	
Oropharynx	23(12/53)	
Paranasal sinuses	8 (4/53)	
Hypopharynx	4(2/53)	
Nasopharynx	4(2/53)	
**Stage**		
EI	4 (2/53)	0.312
EII	15 (8/53)	
EIII	26(14/53)	
EIV	55(29/53)	

* Other HR genotypes: Other high-risk genotypes

** Other LR genotypes: Other low-risk genotypes.

**Table 4.** HPV load and physical status in patients with HNSCC.

Tumour site	Clinical data					Viral load and physical status of HPV-16	
	**T**	**N**	**HPV**	**Copies of E6/β-globin**	**Copies of E2/β-globin**	**E2/E6 ratio**	**Physical status of virus**
Oropharynx	4	2b	16, 18	ND*	0	No E2	Integrated
Larynx	3	0	16	4	0	No E2	Integrated
Larynx	4	3	16	4	0	No E2	Integrated
Oral cavity	4	0	16	4	0	No E2	Integrated
Oropharynx	2	0	16	16	5	0.8	Mixed
Oral cavity	3	0	16	9	0	No E2	Integrated
Oropharynx	4	3	16	20	4	1.75	Episomal
Oral cavity	2	0	16,18,31	ND*	0	No E2 No E6	NA**
Oral cavity	4	2b	16	3	0	No E2	Integrated
Larynx	2	0	6,16	5	0	No E2	Integrated
Oropharynx	3	0	16	2	0	No E2	Integrated
Larynx	4	0	6,11 and 16	3	0	No E2	Integrated
Larynx	2	0	6,11 and 16	8	0	No E2	Integrated
Oropharynx	4	0	11,61	10	7	0.5	Mixed
Oral cavity	2	3	6,11 and 16	ND*	0	No E2 No E6	NA**
Oral cavity	4	2b	11, 16	3	0	No E2	Integrated
Oral cavity	2	1	6,11 and 16	3	0	No E2	Integrated
Hypopharynx	4	2c	6,11 and 16	3	0	No E2	Integrated
Larynx	3	3	6,11 and 16	4	0	No E2	Integrated
Larynx	3	0	6,11 and 16	3	0	No E2	Integrated
Oral cavity	2	0	6,11,16,18	ND*	0	No E2 No E6	NA**
Oral cavity	3	0	6,11 and 16	ND*	0	No E2 No E6	NA**
Nasopharynx	4	3	11, 16, 51	ND*	0	No E2 No E6	NA**
Oral cavity	1	0	6,11,16,53	4	0	No E2	Integrated
Larynx	2	0	6,11 and 16	2	0	No E2	Integrated
Hypopharynx	2	3	6,11,16,31	ND*	0	No E2 No E6	NA**
Oropharynx	2	2b	6,11,16,31	3	0	No E2	Integrated
Larynx	2	0	6,16,18,31	3	0	No E2	Integrated
Oral cavity	2	1	6,16,18,31	4	0	No E2 No E6	NA**
Nasopharynx	3	0	6,11,16,31	ND*	0	No E2 No E6	NA**

ND* Not determined.

NA** Not applicable.
